# etymologia: *Brucella* [broo-sel′ə]

**DOI:** 10.3201/eid1408.089999

**Published:** 2008-08

**Keywords:** etymology, Brucella, David Bruce

Genus of gram-negative, aerobic coccobacilli of the family Brucellaceae, named after Sir David Bruce (1855–1931), a Scottish physician who served abroad with the Royal Army Medical Corps. He investigated Malta fever, a mysterious undulating fever that affected many British soldiers. In 1887 Dr. Bruce established a causal relationship between the disease and an organism later designated *Brucella melitensis* (from Malta). *Brucella* spp. include animal parasites and pathogens, transmissible to humans through dairy products or contact with infected animal tissue.

